# Evaluation of an online advanced suicide prevention training for pharmacists

**DOI:** 10.1007/s11096-023-01636-3

**Published:** 2023-09-13

**Authors:** Samantha Pilbrow, Lexy Staniland, Hannah V. Uren, Fiona Shand, Janey McGoldrick, Emily Thorp, Monique MacKrill, Joanna C. Moullin

**Affiliations:** 1 School of Population Health, Curtin University, Perth, WA Australia; 2 Curtin enAble Institute, Curtin University, Perth, WA Australia; 3 Black Dog Institute, Sydney, NSW Australia; 4 University of New South Wales, Sydney, NSW Australia; 5 Pharmaceutical Society of Australia Tasmanian Branch, Hobart, TAS Australia; 6 The Pharmacy Guild of Australia Tasmanian Branch, Battery Point, TAS Australia

**Keywords:** Community pharmacists, Health education, Suicide prevention

## Abstract

**Background:**

With the pharmacist role extending internationally to include health promotion and harm reduction, pharmacists are well-suited to adopt a frontline role within suicide prevention efforts. To maximise their abilities to implement suicide prevention strategies, suicide prevention training is essential to improve pharmacists’ knowledge of, attitudes towards, and confidence in pharmacy-based suicide prevention.

**Aim:**

This study aimed to evaluate the impact of an online Advanced Suicide Prevention Training for Pharmacists and explore how participant feedback may direct training improvements.

**Method:**

One hundred and fifty pharmacists in Tasmania, Australia, completed the training. Of these, 109 participants completed surveys pre-, post- and 6-months post-training to evaluate changes in suicide prevention knowledge, confidence, and attitudes, and explore participants’ perceptions of the training.

**Results:**

Significant improvements were observed in suicide prevention attitudes (*F*(2, 20) = 4.12, *p* = 0.032, partial η^2^ = 0.292), and self-efficacy (*F*(2, 20) = 7.84, *p* = 0.003, partial η^2^ = 0.439), across the three timepoints, with improvements to knowledge and confidence evident between pre- and post-training (*p* < 0.05). Qualitative data reflected that the training was beneficial in aiding the identification and support of at-risk individuals, however barriers such as the pharmacy setting, personal discomfort, and perceptions of the pharmacist role were identified as impeding the implementation of suicide prevention within pharmacy practice.

**Conclusion:**

Training is an effective means of improving pharmacists’ suicide prevention knowledge, confidence, and attitudes. While personal barriers to suicide prevention improved, contextual and social barriers continue to impede pharmacists’ implementation of suicide prevention in practice.

**Supplementary Information:**

The online version contains supplementary material available at 10.1007/s11096-023-01636-3.

## Impact statements

- Improved pharmacist suicide prevention knowledge, confidence, and attitude may positively influence pharmacists’ abilities to identify at-risk patients and initiate potentially life-saving suicide preventative action within their practice.
- In providing training in an online format, up-scaling pharmacist-focused suicide prevention training to a greater population may increase the uptake of suicide prevention behaviours by pharmacists within the pharmacy setting.
- Pharmacists identified contextual and social barriers that impede suicide prevention behaviours with the pharmacy setting, suggesting that while pharmacist may intend to implement these behaviours, doing so remains challenging.

## Introduction

The need for effective suicide prevention interventions is evident, with over 700,000 people dying by suicide globally each year [1]. Addressing suicide requires a multidisciplinary approach, and it has been argued that pharmacists are well positioned to engage in suicide prevention due to their high accessibility and frequent interactions with individuals at risk of suicide [2–6]. These interactions allow rapport and trust to develop, creating psychological safety for disclosure of mental health difficulties and suicidal thoughts [2, 7]. The frequency of interactions also enables pharmacists to observe warning signs of suicidality, including heightened stress, and changes to mood, behaviour, and medications patterns [2, 8, 9]. By identifying these warning signs, pharmacists can implement suicide prevention by providing preliminary support creating a pathway to safety [2, 10]. As such, training pharmacists with suicide prevention skills as a form of primary prevention has been highlighted as promising yet underutilised [2, 7, 10–12].

Despite the potential for pharmacists to be more active in suicide prevention, implementation of these practices is not without barriers [10]. Studies suggest that some pharmacists feel unprepared to assume a frontline role in suicide prevention [5, 13, 14]. In turn, training initiatives have increased; however, the long-term effectiveness these initiatives remain unclear [15–18]. Furthermore, many training programs accessible to pharmacists focus on general mental health awareness, limiting the potential for such programs to improve suicide prevention knowledge [4, 19–21]. Without specific suicide prevention training, pharmacists’ abilities to offer appropriate support is limited, and likely contributes to a reluctance to engage in suicide prevention strategies [22].

The Black Dog Institute (BDI), a leading Australian suicide prevention organisation, developed the Advanced Suicide Prevention Training for Pharmacists [23]. Accredited by the Pharmaceutical Society of Australia, the training involves psychoeducation activities specifically relevant to practicing pharmacists and the pharmacy setting.

### Aim

This study aimed to evaluate the impacts of the Advanced Suicide Prevention Training for Pharmacists on pharmacists’ knowledge of, confidence in, and attitudes towards suicide prevention over time, and understand the training’s practical utility, and potential implementation barriers and facilitators to inform training improvements.

### Ethics approval

Approval for this study was obtained from Curtin University’s Research Ethics Committee (3 June 2020; HREC2020-0106).

## Method

A cross-sectional design comprising an online survey of quantitative measures, with short answer questions was used. A pre-post-follow-up approach was employed to analyse data collected from the online training sessions of 5 participant groups across 2020, 2021, and 2022.

### Participants

A convenience sample of Tasmanian pharmacists was recruited through official communications from the BDI and other Australian pharmaceutical associations. Eligible participants were pharmacists currently practicing and residing within Tasmania, Australia.

### Procedure

To participate, pharmacists enrolled using a link in the marketing materials. Prior to the training, all enrolled pharmacists received access to the pre-training survey via email. This included an information sheet and consent form. Consenting participants completed a 15-minute survey and were provided with a support services resource sheet. Regardless of research participation, all enrolled pharmacists received the training for free, with no differences in the content. After completing their training session, participants repeated this process with the post-training and 6-months post-training surveys.

### Intervention

Following a face-to-face pilot study in Hobart, the training was trialled using online delivery, in response to COVID-19. Training modifications were made based on participant feedback. The Advanced Suicide Prevention Training for Pharmacists comprises a 2.5-hour workshop delivered by clinical psychologists from the BDI. All participants in this study completed the online version, via Zoom. The training encompasses 6 learning outcomes, including understanding the prevalence of suicide, identifying risk factors and warning signs of suicidality, practising conversations with someone at-risk of suicide, and increasing awareness of available resources and referral pathways to keep someone safe. Key training activities included analysing patient case scenarios, interactive role-plays, and an exemplar video illustrating how to implement learned knowledge within a pharmacy setting. Each training component was paired with debriefs, allowing participant discussion and shared learning. To help consolidate their learning, participants received a digital workbook to write in and retain after the training.

### Measures

Demographic information was collected regarding participants’ age, gender, preferred language, and whether they identified as Aboriginal and/or Torres Strait Islander. Regarding pharmacy background, participants indicated their level of pharmacy work experience, current practice setting, and if they had previously participated in suicide prevention training.

To assess changes in suicide prevention knowledge, confidence, and self-efficacy, measures were designed by the BDI, in conjunction with the research team (see Supplementary Materials). Suicide Prevention Knowledge and Confidence were each assessed with a single item at each timepoint, rated on a 10-point Likert scale, from 1 (not at all) to 10 (extremely) knowledgeable or confident. Suicide prevention knowledge was further assessed through a scenario-based measure in which participants were presented with 5 scenarios describing an example patient interaction (e.g., *“I feel like I might do something to myself … I’ve been thinking about suicide”*). Participants were asked to rate 2 opposing responses to the scenarios on a scale from 1 (highly inappropriate) to 7 (highly appropriate). Scores of appropriate and inappropriate responses were then summed separately. Total scores could range from 5 to 35, with high scores of appropriate responses and lower scores of inappropriate responses reflecting higher suicide prevention knowledge.

Suicide prevention self-efficacy was assessed using a 6-item measure where participants rated their agreement with a series of statements (e.g., *“I am able to recognise signs that someone may be experiencing suicidal thoughts”*), on a 5-point Likert scale. Responses ranged from 1 (strongly disagree) to 5 (strongly agree), with total scores ranging from 6 to 30. Higher scores indicated greater suicide prevention self-efficacy. Internal consistency was acceptable across the pre-training (α = 0.86), post-training (α = 0.80), and 6-months post-training (α = 0.77) surveys.

Attitudes towards suicide prevention were assessed using 10 items of an adapted version of the Attitudes to Suicide Prevention Scale (ASPS; e.g., *“I resent being asked to do more about suicide”*), rated on a 5-point Likert scale, ranging from 1 (strongly disagree) to 5 (strongly agree), with lower scores indicating more positive attitudes towards suicide prevention [24]. Participants were also asked: *“What proportion of suicides do you consider preventable?”*, with responses made on a 5-point Likert scale from 1 (none) to 5 (all). The ASPS has demonstrated acceptable internal consistency (α = 0.77) [24]. Internal consistency of this version was acceptable across pre-training (α = 0.73), post-training (α = 0.90), and 6-months post-training (α = 0.77).

In the post-training surveys, 5 items assessed participant’s perspectives on implementing suicide prevention into pharmacy practice (e.g., *“It is feasible”*), rated on a 5-point Likert scale, ranging from 1 (strongly disagree) to 5 (strongly agree). 4 open-ended questions were included to gauge areas for training improvement and perceptions regarding the barriers and facilitators faced by pharmacists when identifying and initiating conversations with individuals at risk of suicide.

### Data analysis

Data from the 3 timepoints were deidentified, cleaned and case matched. SPSS was used for assumptions testing and analyses. Bivariate correlations assessed for covariates between age, gender, registration length, previous suicide training, and all outcome variables. Previous suicide prevention training significantly correlated with 6 outcome variables (*p* < 0.05) and was therefore included as a covariate in subsequent inferential tests.

Changes in the pharmacists’ suicide prevention knowledge, confidence, and attitudes were assessed using repeated measures ANOVAs on the following outcome variables: suicide prevention self-efficacy, attitudes towards suicide prevention, the total sum of appropriate and inappropriate responses to example patient comments, and the average proportion of suicides viewed as preventable, across each timepoint. Single-item measures of knowledge and confidence were excluded due to insufficient 6-months post-training data. Paired samples *t*-tests were conducted (α = 0.05) to compare mean differences in scores on the outcome variables across the pre- and post-training timepoints, and to compare mean differences in scores on the pharmacist intervention statements across the post-training and 6-months post-training timepoints.

Open-ended responses were analysed using inductive-deductive conventional content analysis to explore participant feedback [25]. Triangulation was achieved through researcher discussion, and reflexive journalling was conducted.

## Results

Across the 5 participant groups, 150 pharmacists completed the training. 109 participants completed at least 1 of the 3 surveys, 96 completed the pre-training survey, 55 completed the post-training survey, and 26 completed the 6-months post-training survey. Twelve participants completed all 3 surveys. Participants’ age ranged from younger than 25 to older than 65, with majority identifying as female (75%). Aligning with the target population, most participants were community pharmacists (64.2%). 55 participants (58.5%) reported no previous participation in suicide prevention training. Demographic data are presented in Table 1.

**Table 1:** Participant demographics

Variable	*n* (%)
Gender	
Female	72 (75)
Male	24 (25)
Transgender Female	0
Transgender Male	0
Other (*please specify*)	0
Age (in years)	
< 25	19 (19.8)
25–34	31 (32.3)
35–44	19 (19.8)
45–54	13 (13.5)
55–64	10 (10.4)
> 65	4 (4.2)
Prefer not to say	0
Identifying as aboriginal and/or torres strait islander	
Yes	2 (2.1)
No	94 (97.9)
Prefer not to say	0
Pharmacy role	
Community pharmacist	61 (64.2)
Hospital pharmacist	12 (12.6)
Pharmacist in a general practice	2 (2.1)
Academia/research	3 (3.2)
Consulting pharmacist	1 (1.1)
Pharmacy assistant	2 (2.1)
Pharmacy technician	3 (3.2)
Other*	11 (11.6)
Location of pharmacy	
Capital city CBD/Inner city	18 (19.8)
Capital city suburbs	29 (31.9)
Rural city (Population equal to or greater than 100,000)	13 (14.3)
Rural (Population between 5001 to 99,999)	23 (25.3)
Remote (Population less than or equal to 5000)	8 (8.8)
Length of registration (in years)	
0–4	38 (40.0)
5–9	16 (16.8)
10–20	22 (23.2)
> 20	19 (20.0)
Previous suicide prevention training	
Yes	9 (41.5)
No	55 (58.5)

*Included Department of Health (1), Government and non-Government organisations (1), non-Pharmacist peak body representative (1), Opioid pharmacotherapy Pharmacist (1), Pharmacist working in aged care (1), Pharmacist working in the private sector (1), Intern Pharmacists across community (2), and hospital (2) settings, and Pharmacy student currently working as a Pharmacy assistant (1)

Repeated measures ANOVA analyses indicated that participants’ suicide prevention self-efficacy significantly improved across timepoints (*F*(2,20) = 20.48, *p* < 0.001, partial η^2^ = 0.672), and remained significant after adjusting for previous suicide prevention training (*F*(2, 20) = 7.84, *p* = 0.003, partial η^2^ = 0.439). Attitudes towards suicide prevention also significantly improved across timepoints (*F*(2, 20) = 5.03, *p* = 0.017, partial η^2^ = 0.335), remaining significant after adjusting for the covariate (*F*(2, 20) = 4.12, *p* = 0.032, partial η^2^ = 0.292). Differences in the appropriateness and inappropriateness of participants’ responses to suicide prevention scenarios across the 3 timepoints were non-significant (*F*(2, 20) = 0.43, *p* = 0.656, partial η^2^ = 0.041; *F*(2, 20) = 0.60, *p* = 0.559, partial η^2^ = 0.056). These differences remained non-significant after adjusting for previous suicide prevention training (*F*(2, 20) = 0.17, *p* = 0.844, partial η^2^ = 0.017; *F*(2, 20) = 0.73, *p* = 0.496, partial η^2^ = 0.068). The difference in the proportion of suicides perceived as preventable by participants across the 3 timepoints was also non-significant before (*F*(2, 20) = 1.62, *p* = 0.222, partial η^2^ = 0.140), and after (*F*(2, 20) = 0.66, *p* = 0.528, partial η^2^ = 0.062), adjusting for previous training. Pairwise comparisons are presented in Table 2.

**Table 2:** Repeated measures ANOVA pairwise comparisons of the variables of interest across pre-training, post-training, and 6-months post-training timepoints

	Pairwise comparisons (*n* = 12)		
	*M*	95% CI	*p*
Appropriate responses			
Pre-training	27.50	[24.90, 30.12]	Pre- vs. Post-training: *p* = 0.010
Post-training	30.25	[27.88, 32.62]	Post-training vs. Follow-up: *p* = 0.135
Follow-up	29.24	[27.18, 31.30]	Follow-up vs. Pre-training: *p* = 0.238
Inappropriate responses			
Pre-training	14.00	[11.90, 16.10]	Pre- vs. Post-training: *p* = 1.000
Post-training	14.83	[12.10, 17.57]	Post-training vs. Follow-up: *p* = 0.353
Follow-up	13.24	[10.63, 16.21]	Follow-up vs. Pre-training: *p* = 1.000
Self-Efficacy			
Pre-training	19.33	[16.81, 21.86]	Pre- vs. Post-training: *p* < 0.001
Post-training	25.25	[23.60, 26.91]	Post-training vs. Follow-up: *p* = 0.338
Follow-up	24.25	[22.44, 26.06]	Follow-up vs. Pre-Training: *p* < 0.001
Attitudes			
Pre-training	17.75	[15.29, 20.21]	Pre- vs. Post-training: *p* = 0.040
Post-training	14.67	[11.86, 17.48]	Post-training vs. Follow-up: *p* = 0.402
Follow-up	16.67	[14.52, 18.81]	Follow-up vs. Pre-training: *p* = 0.394
Perceived preventability			
Pre-training	3.92	[3.47, 4.36]	Pre- vs. Post-training: *p* = 0.424
Post-training	4.33	[3.91, 4.76]	Post-training vs. Follow-up: *p* = 1.000
Follow-up	4.42	[4.00, 4.83]	Follow-up vs. Pre-training: *p* = 0.180

*M *mean, *CI* confidence interval

Paired samples *t*-test analyses indicated that participants’ knowledge, confidence, suicide prevention self-efficacy, and attitudes towards suicide prevention significantly improved between pre-training and post-training, as did the number of appropriate responses (*p* < 0.05; see Table 3). Differences in inappropriate responses and the proportion of suicides viewed as preventable were non-significant (*p* > 0.05). Paired samples *t*-tests conducted on the pharmacist intervention statements revealed that participants’ perceptions of support, feasibility, and appropriateness significantly decreased between post-training and 6-months post-training (*p* < 0.05; see Table 4). Differences in participants’ perceptions of acceptability to other healthcare professionals and patients were non-significant (*p* > 0.05).

**Table 3:** Paired samples t-tests examining differences in the variables of interest across pre-training and post-training timepoints

	Pre-training (*n* = 45)		Post-training (*n* = 45)		Mean difference	95% CI of the difference	*t*	df	Sig. (2-tailed)	*d*	95% CI
	*M*	*SD*	*M*	*SD*							
Knowledge	4.53	2.00	7.76	0.98	3.22	[2.69, 3.76]	12.13	44	< 0.001	1.78	[1.32, 2.28]
Appropriate responses	25.96	3.61	29.67	3.28	3.71	[2.82, 4.60]	8.41	44	< 0.001	2.96	[− 1.64, − 0.86]
Inappropriate responses	13.67	3.66	14.18	4.86	0.51	[− 0.48, 1.50]	1.04	44	0.303	3.29	[− 0.45, 0.14]
Confidence	4.27	2.07	7.31	1.38	3.04	[2.54, 3.55]	12.07	44	< 0.001	1.70	[1.32, 2.27]
Self-efficacy	17.82	4.58	24.29	2.64	6.47	[5.00, 7.94]	8.87	44	< 0.001	4.89	[− 1.72, − 0.92]
Attitudes	20.44	4.63	16.71	5.00	3.73	[− 4.90, − 2.57]	− 6.46	44	< 0.001	3.88	[0.61, 1.31]
Perceived preventability	3.91	0.82	4.13	0.73	0.22	[− 0.04, 0.49]	1.70	44	0.096	0.88	[− 0.55, 0.05]

Appropriate Responses = Total sum of Appropriate Responses, Inappropriate Responses = Total sum of Inappropriate Responses, Self-Efficacy = Total scores on the Suicide Prevention Self-Efficacy Scale, Attitudes = Total scores on Attitudes towards Suicide Prevention Scale, Perceived Preventability = The proportion of suicides viewed as preventable. *M* = Mean, *SD* = Standard deviation, CI = Confidence interval

**Table 4:** Paired samples t-tests examining differences in responses to pharmacist intervention statements regarding the implementation of suicide prevention, across post-training and 6-month follow-up timepoints

Intervention statements	Post-training (*n* = 17)		Six-months post-training (*n* = 17)		Mean difference	95% CI of the difference	*t*	df	Sig. (2-tailed)	*d*	95% CI
	*M*	*SD*	*M*	*SD*							
“I am supportive”	4.82	0.39	4.59	0.51	0.24	[− 0.46, − 0.01]	− 2.22	16	0.041	0.44	[− 1.04, − 0.02]
“It is feasible”	4.35	0.79	4.06	0.75	0.29	[− 0.54, − 0.05]	− 2.58	16	0.020	0.47	[− 1.14, − 0.96]
“It is appropriate”	4.94	0.25	4.56	0.13	0.36	[− 0.64, − 0.11]	− 3.00	15	0.009	0.50	[− 1.30, − 0.18]
“It would be acceptable to other healthcare professionals”	4.00	0.79	3.71	0.85	0.29	[− 0.83, 0.24]	− 1.16	16	0.264	1.05	[− 0.76, 0.21]
“It would be acceptable to patients”	4.47	0.80	4.18	0.53	0.29	[− 0.69, 0.10]	− 1.57	16	0.136	0.77	[− 0.87, 0.11]

*M* = Mean, *SD* = Standard deviation, *CI* = Confidence interval

### Open-ended responses: Pharmacist feedback

Participants provided short answers to questions related to what aspects of the training were helpful or could be improved. Pharmacy-specific sections of the training were viewed as helpful. Specifically, identifying warning signs of suicidality, how to initiate conversations about suicidality, and the video demonstrating how pharmacists might implement these practices with a patient. Participants also valued the ability to share experiences and approaches to suicide prevention. One participant described that “*the personal approach allowed attendees to feel welcome and realise that not everyone is an expert.”*

In comparison, some participants felt the content could be further tailored to address the pharmacist role in suicide prevention and suggested including more example scenarios to capture patients’ differing circumstances. Additionally, participants wanted clearer steps to follow once an at-risk patient is identified. With practising conversations and role-playing highlighted as beneficial, participants recommended that the time spent outlining the support services available to pharmacists could be supplemented with take-home materials and reallocated to practical components.

Improvements were also suggested regarding the delivery format and the number of training sessions provided. Some participants found the online format “*challenging,”* describing that the format may negatively impact participants’ abilities to role play and share knowledge. Participants advised additional training sessions, with the benefits of this captured by this response: “*A follow-up [training] will reinforce how others are applying this knowledge … The more we do this, the better we will be.”*

### Pharmacists’ perceived barriers and facilitators to identifying and initiating conversations

To guide ongoing training development, participants provided their perspectives on the barriers and facilitators to identifying at-risk individuals and initiating conversations surrounding suicidality. The key barriers identified were the pharmacy setting, personal discomfort, and societal perceptions of pharmacists’ role in suicide prevention. Participants described various constraints within the pharmacy setting, including excessive workloads, understaffing, and inadequate compensation, privacy, and time to conduct conversations about suicide with patients. Without these resources, participants felt it could be inappropriate to initiate or could compromise the quality of these conversations: “*there is a risk that pharmacists will have other workplace pressures leading to less-than-optimal conversations.”* Furthermore, personal discomfort deterred participants from initiating conversations. One participant reflected: “*Personal discomfort hidden behind excuses, such as too busy, not the right time… Not being confident that you will be able to successfully navigate the conversation*.” Participants appeared to relate their personal discomfort to a lack of training and experience in initiating conversations surrounding suicidality, suggesting that with training, comfort to initiate conversations about suicide could be improved.

While pharmacists’ confidence and skills were key to engaging in conversations about suicide with patients, societal perceptions of whether this is within a pharmacist’s scope of practice was an identified barrier. They shared that some patients did not categorise pharmacists as trusted health professionals and, therefore, may be reluctant to disclose personal information, making conversations regarding suicidality difficult. Additionally, some participants described that their role within suicide prevention was not respected by other healthcare professionals. One participant stated, “*we need other health professionals to accept the idea that pharmacists are an acceptable go between.*” As such, participants felt greater collaboration between healthcare professionals was necessary to fortify the relationships between healthcare professionals and strengthen the support networks available to patients. Participants were hopeful for more collaborative relationships with healthcare professionals in future, noting *“Qualifications aside, we are all community.”*

Participants identified positive patient-professional relationships and adequate training/education as key facilitators to identifying at-risk individuals. Participants emphasised the importance of developing rapport with patients. Once established, they suggested that this enabled conversation openness, facilitating changes in patients’ behaviour to be observed and more confident conversations regarding suicidality. One participant shared that *“training has been essential to give confidence in approaching the issue* [*suicide*].” Participants also expressed that training had facilitated behaviour change, such as directly asking individuals about their mental health and thoughts of suicide.

## Discussion

The Advanced Suicide Prevention Training for Pharmacists demonstrated significant and lasting improvements in pharmacists’ suicide knowledge, suicide prevention confidence and self-efficacy, and suicide prevention attitudes. Participants’ feedback emphasised the contextual strengths of the training, and barriers to its efficacy. In particular, they noted the inclusion of suicidality terminology and the warning signs of suicidality particularly useful in improving knowledge, enabling them to identify at-risk patients and initiate conversations about suicide [26]. Participants also appreciated the exemplar video depicting the implementation of suicide prevention skills and noted the importance of role-playing scenarios and dedicated practise time in strengthening their confidence in applying the learned skills [10]. Applying Social Cognitive Theory [27], which argues that learning new skills is both cognitive and social, role-play components enable participants to first observe, and then practise initiating a conversation about suicide with at-risk patients [10, 28].

### Implications for pharmacy practice

Our findings demonstrate the practical utility of training in assisting pharmacists to identify at-risk individuals and provide potentially life-saving support [28]. While suicide prevention knowledge, confidence, and attitudes are influential factors for enacting suicide prevention behaviours [29, 30], addressing these factors alone may not be sufficient to overcome practice-related barriers [10, 31]. Consistent with previous work [5, 10, 15, 32–34], various contextual and social barriers were identified, including insufficient time, resources, and confidence. Therefore, while post-training improvements may foster pharmacists’ intentions to enact suicide prevention behaviours, the barriers may create a gap between intentions and the implementation of these behaviours within pharmacy practice [30].

### Directions for training development

This training is one of the first programs delivered entirely online, whilst also effectively improving pharmacists’ suicide prevention knowledge, confidence, and attitudes towards identifying and initiating conversations with at-risk individuals. While online training may not be preferred by all, its cost-effectiveness and accessibility make it advantageous for reaching pharmacists in remote areas or facing barriers to attending face-to-face sessions.

Further development may be necessary to enhance improvements and better address participants’ needs. With the current study indicating no progress at the follow-up assessment, strategies including spaced learning, skill-building workshops, or additional training sessions may be needed to refresh participants’ knowledge and skills [28, 35]. These strategies could also address the decline in pharmacists’ perceptions of incorporating suicide prevention behaviours in pharmacy practice. Furthermore, participants’ requests for more practise time and pharmacist specific content highlights the value of incorporating additional skill building activities. Including a diverse range of scenarios contextualised within the pharmacy setting may also increase the training’s practical utility, as this can allow participants to relate the scenarios to their personal experiences, strengthening their learning [36]. Adopting an interprofessional component may address concerns regarding how other healthcare professionals perceive the pharmacist’s role in suicide prevention, facilitating effective referral pathways and multidisciplinary support [37, 38].

### Limitations

Due to low participation numbers and the impact of COVID-19 on pharmacists’ responsibilities, this study faced limitations in investigating the training effects over time. The limited number of participants who completed all surveys may introduce self-selection bias, as it is unlikely the sample is well-representative of the pharmacist population in Australia [39]. These factors, along with high attrition rates, are not unexpected in a longitudinal design but should be acknowledged. Excluding the ASPS, all other measures were developed by the BDI or research team, and their psychometric properties have not yet been evaluated, thus requiring validation for future research.

### Directions for future research

Future research should investigate the relationship between pharmacists’ suicide prevention knowledge, confidence, and attitudes, and their implementation of suicide prevention behaviours in pharmacy practice. Comparing the effectiveness of online training with face-to-face formats would be valuable, considering the economic benefits and accessibility of the online format. The effectiveness in diverse samples should be examined. It is recommended that the training is scaled-up, with evaluation conducted on a larger sample size.

## Conclusion

The Advanced Suicide Prevention Training for Pharmacists effectively improves pharmacists’ knowledge of, confidence in, and attitudes towards identifying and initiating conversations with at-risk individuals. Despite these positive outcomes, structural and cultural barriers remain, which require further exploration and efforts to address.

### Electronic supplementary material

Below is the link to the electronic supplementary material.

Supplementary Material 1 (DOCX 32 kb)
