# Supplementary material for: Evaluation of an online advanced suicide prevention training for pharmacists
Source: Int J Clin Pharm. 2023 Sep 13;45(5):1203–11. doi: 10.1007/s11096-023-01636-3 (PMC10600280; doi:10.1007/s11096-023-01636-3)
Supplement: Supplementary file 1 — Supplementary Material 1 (DOCX 32 kb) [file 11096_2023_1636_MOESM1_ESM.docx]

**Measures**

**Suicide Prevention Knowledge**

Rate your**knowledge** with regard to recognising and having a conversation on suicidality

|  |  | |  | |  | |  | |  | |  | |  | |  | |  |
| --- | --- | --- | --- | --- | --- | --- | --- | --- | --- | --- | --- | --- | --- | --- | --- | --- | --- |
| 1 | 2 | | 3 | | 4 | | 5 | | 6 | | 7 | | 8 | | 9 | | 10 |
| **Not at all knowledgeable** | |  | |  | |  | |  |  |  | |  | |  | | **Extremely knowledgeable** | |

For each of the following patient comments, please rate the appropriateness of each response.

1. **"I feel like I might do something to myself... I’ve been thinking about**

|  | **-3**  **Highly inappropriate response** | **-2 Inappropriate response** | **-1**  **Marginally inappropriate response** | **0**  **Neither appropriate nor inappropriate** | **+1**  **Marginally appropriate** | **+2 Appropriate response** | **+3**  **Highly appropriate response** |
| --- | --- | --- | --- | --- | --- | --- | --- |
| a. You say you’re suicidal, but what is really bothering you? |  |  |  |  |  |  |  |
| b. Can you tell me more about your suicidal feelings? |  |  |  |  |  |  |  |

1. **"I’m so lonely, so tired. There just isn’t anywhere left to turn"**

|  | **-3**  **Highly inappropriate response** | **-2 Inappropriate response** | **-1**  **Marginally inappropriate response** | **0**  **Neither appropriate nor inappropriate** | **+1**  **Marginally appropriate** | **+2 Appropriate response** | **+3**  **Highly appropriate response** |
| --- | --- | --- | --- | --- | --- | --- | --- |
| a. You seem so alone, so miserable. Have you been feeling suicidal? |  |  |  |  |  |  |  |
| b. Come on now. Things can’t be all that bad. |  |  |  |  |  |  |  |

1. **"I don’t think there’s really anyone who cares whether I’m alive or dead"**

|  | **-3**  **Highly inappropriate response** | **-2 Inappropriate response** | **-1**  **Marginally inappropriate response** | **0**  **Neither appropriate nor inappropriate** | **+1**  **Marginally appropriate** | **+2 Appropriate response** | **+3**  **Highly appropriate response** |
| --- | --- | --- | --- | --- | --- | --- | --- |
| a. It sounds like you’re feeling pretty isolated |  |  |  |  |  |  |  |
| b. Why do you think that no one cares about you anymore? |  |  |  |  |  |  |  |

1. **"How could you ever help me? Have you ever wanted to kill yourself?"**

|  | **-3**  **Highly inappropriate response** | **-2 Inappropriate response** | **-1**  **Marginally inappropriate response** | **0**  **Neither appropriate nor inappropriate** | **+1**  **Marginally appropriate** | **+2 Appropriate response** | **+3**  **Highly appropriate response** |
| --- | --- | --- | --- | --- | --- | --- | --- |
| a. It sounds like you’re concerned about whether I can understand and help you |  |  |  |  |  |  |  |
| b. Sure, I’ve thought about suicide sometimes. But I always found more realistic solutions to my problems |  |  |  |  |  |  |  |

1. **"My family is financially well off, and my husband spends plenty of time with me, even though he has a successful law career. Even my kids have been doing well. They get good marks at school and have lots of free time activities with their friends. But nothing seems to interest me. Life is just a bore…"**

|  | **-3**  **Highly inappropriate response** | **-2 Inappropriate response** | **-1**  **Marginally inappropriate response** | **0**  **Neither appropriate nor inappropriate** | **+1**  **Marginally appropriate** | **+2 Appropriate response** | **+3**  **Highly appropriate response** |
| --- | --- | --- | --- | --- | --- | --- | --- |
| a. Considering all you have going for you, your problems can’t be all that serious. Try to focus more on the positive aspects of your situation |  |  |  |  |  |  |  |
| b. So even though things seem to be going well at one level, life still seems pretty depressing, even if its hard to say exactly why |  |  |  |  |  |  |  |

**Suicide Prevention Confidence**

Rate your **confidence** with regard to recognising and having a conversation on suicidality

|  |  | |  | |  | |  | | |  | |  | |  | |  | |  |
| --- | --- | --- | --- | --- | --- | --- | --- | --- | --- | --- | --- | --- | --- | --- | --- | --- | --- | --- |
| 1 | 2 | | 3 | | 4 | | 5 | | | 6 | | 7 | | 8 | | 9 | | 10 |
| **Not at all confident** | |  | |  | |  | |  |  | |  | |  | |  | | **Extremely confident** | |

**Suicide Prevention Self-Efficacy**

Please rate your agreement with the following statements

|  | **Strongly disagree** | **Disagree** | **Neither agree nor disagree** | **Agree** | **Strongly agree** |
| --- | --- | --- | --- | --- | --- |
| I am able to recognise signs that someone may be having thoughts of suicide |  |  |  |  |  |
| If someone was showing signs of suicide, I would directly raise the question of suicide with them |  |  |  |  |  |
| I have the skills to have a conversation with someone about their thoughts of suicide |  |  |  |  |  |
| I have the skills to intervene safely and constructively with someone considering suicide |  |  |  |  |  |
| I can work together with someone to keep them safe from suicide |  |  |  |  |  |
| I know how to support a person to get professional help if they are having thoughts of suicide |  |  |  |  |  |

**Attitudes towards Suicide Prevention^1^**

Please rate your agreement with the following statements

|  | **Strongly disagree** | **Disagree** | **Neither agree nor disagree** | **Agree** | **Strongly agree** |
| --- | --- | --- | --- | --- | --- |
| I resent being asked to do more about suicide |  |  |  |  |  |
| Suicide prevention is not my responsibility |  |  |  |  |  |
| Making more funds available to the appropriate health services would make no difference to the suicide rate |  |  |  |  |  |
| If people are serious about committing suicide, they don’t tell anyone |  |  |  |  |  |
| If a person survives a suicide attempt, then this was a ploy for attention |  |  |  |  |  |
| People have the right to take their own lives |  |  |  |  |  |
| Since unemployment and poverty are the main causes of suicide, there is little that an individual can do to prevent it |  |  |  |  |  |
| I don’t feel comfortable assessing someone for suicide risk |  |  |  |  |  |
| Suicide prevention measures are a drain on resources, which would be more useful elsewhere |  |  |  |  |  |
| There is no way of knowing who is going to commit suicide |  |  |  |  |  |

What proportion of suicides do you consider preventable?

|  |  |  |  |  |
| --- | --- | --- | --- | --- |
| 1 | 2 | 3 | 4 | 5 |
| **None** |  |  |  | **All** |

1. Herron J, Ticehurst H, Appleby L, Perry A, Cordingley L. Attitudes toward suicide prevention in front-line health staff. *Suicide Life Threat Behav*. 2001;31(3):342-347. doi:10.1521/SULI.31.3.342.24252

**Perceived Training Effectiveness and Practical Utility**

Please rate the extent to which you agree with each of the following statements regarding pharmacists/pharmacy assistants identifying and initiating conversations with patients after a suicide attempt or thoughts of suicide?

|  | **Strongly disagree** | **Somewhat disagree** | **Neither agree nor disagree** | **Somewhat agree** | **Strongly agree** |
| --- | --- | --- | --- | --- | --- |
| I am supportive |  |  |  |  |  |
| It is feasible |  |  |  |  |  |
| It is appropriate |  |  |  |  |  |
| It would be acceptable to other healthcare professionals |  |  |  |  |  |
| It would be acceptable to patients |  |  |  |  |  |

**Open-Ended Questions**

1. Please tell us which sections of the training you found most useful?
2. Please tell us which sections of the training you feel could be improved?
3. What are some of the barriers to pharmacists/pharmacy assistants identifying and initiating conversations with patients after a suicide attempt or thoughts of suicide?
4. What are some of the factors that have helped you to identify and initiate conversations with patients after a suicide attempt or thoughts of suicide?
